# Melt-Electrospun Polyethylene Nanofiber Obtained from Polyethylene/Polyvinyl Butyral Blend Film

**DOI:** 10.3390/polym12020457

**Published:** 2020-02-16

**Authors:** Mohammad Zakaria, Kanta Shibahara, Koji Nakane

**Affiliations:** 1 Frontier Fiber Technology and Science, University of Fukui, Fukui 910-8507, Japan; zakariate@duet.ac.bd (M.Z.); 1sanka305@gmail.com (K.S.); 2 Department of Textile Engineering, Dhaka University of Engineering & Technology, Gazipur 1700, Bangladesh

**Keywords:** polyethylene, nanofiber, melt-electrospinning, crystallinity, blends

## Abstract

We prepared low-density polyethylene (LDPE) nanofiber, a few hundred nanometers in diameter, using polyvinyl butyral (PVB) and a laser melt-electrospinning (M-ESP) device. We blended PVB with LDPE via an internal melt mixer, removed the PVB after M-ESP by ethanol treatment, and studied the influence of PVB on fiber diameter. A substantial diameter reduction with improved crystallinity of LDPE fiber was observed with increased PVB content in the blend. PVB inclusion also increased the polarity of the LDPE/PVB blend, resulting in better spinnability. The removal of PVB from LDPE/PVB blend fiber caused a massive drop in the LDPE fiber diameter, due to fiber splitting, particularly in PVB-rich samples. Fourier transform infrared (FTIR) spectroscopy of fibers confirmed that the prepared nanofiber was the same as pure LDPE fiber.

## 1. Introduction

Electrospinning is an electrohydrodynamic process that draws super-fine continuous fibers using electrostatic force to collect the liquid jet from a polymer solution or melt. This process is widely used to fabricate nanofibers from 50 or more varieties of polymers, due to its simplicity and suitability [1,2]. It has two primary divisions, i.e., solution and melt-electrospinning [2]. Solution electrospinning is an effective and versatile technique [3]. However, the melt-electrospinning (M-ESP) system ensures a safer environment and avoids the use of solvents, including their removal or recycling. M-ESP also produces an extreme loss of mass during solvent evaporation, which ensures better surface smoothness and mechanical properties of the fiber [4]. Moreover, polyolefins such as polypropylene (PP) and polyethylene (PE) have no appropriate solvents at room temperatures [5]. Thus, M-ESP is considered to be a more ecological, productive, cost-effective, and safer alternative to solution electrospinning [5,6,7].

However, relatively few studies have been conducted on electrospinning directly from polymer melts [4,5,6,7,8,9], owing to the high-temperature setup needed for M-ESP systems and the low conductivity and high viscosity of polymer melts. In addition, M-ESP manufactures relatively coarser fiber than solution electrospinning [4]. Using a CO_2_ laser heating source, we introduce a new electrospinning technique that simplifies the high-temperature setup of M-ESP [10]. Our laser melting system eliminates the need for a conventional reservoir for molten polymer, resulting in nozzle-free efficient electrospinning with lower energy consumption [11,12,13,14]. The thermal degradation of the polymer melt caused by long-term heating is also avoided by highly controlled and localized laser heating [15]. Furthermore, to improve polymer conductivity and reduce melt viscosity, multi-component systems such as blends [13,16] and additives [9,17] have been employed for M-ESP with limited success.

Polyethylene (PE) has some outstanding characteristics, including chemical resistance, ease of processing, near-zero moisture absorption, toughness, electrical properties, low friction coefficient, and abrasion resistance, thus ensuring that it constitutes the world’s largest polymer volume consumption [18] [19,20]. However, the low strength and thermal conductivity of bulk PE greatly hinder its functionality [18]. Instead, a significant enrichment of the mechanical and thermal resistivity of polymers has been reported by the proper alignment of polymer chains [21,22]. Therefore, the stretching of PE from bulk into thin films, or micro- or nanofibers, improves the polymer chain arrangement and results in improved mechanical and thermal properties [23,24]. Moreover, in the case of polymeric fiber, diameter conversion from micrometers to nanometers tremendously enhances the specific surface area, producing an excellent smooth covering against ultra-fine particles and microorganisms [2].

Sheng et al. have reported ultra-drawn high-quality PE nanofibers with a diameter of only 50–500 nm, using the two-stage heating of polymer gel [18]. Moreover, PE nanofibers around 300 nm in diameter have been produced by solution-type electrospinning via a high-temperature solution and rotating collector [25]. Li et al. [26] introduced a two-stage solution-gel heating method and developed single PE nanofibers only a hundred nanometers in diameter. Again, however, there have been few attempts to produce PE nanofiber using M-ESP. Rongjian et al. demonstrated a simple M-ESP device but were unable to produce PE fiber less than 5 µm [4] in diameter. No PE fiber less than 1 µm in average diameter has yet been reported, using M-ESP. In our laboratory, nanofibers were successfully obtained using the laser M-ESP system from various polymers with polar groups, namely ethylene-vinyl alcohol copolymer (EVOH) [11,13,14], polylactic acid (PLA) [10], and nylon 6/12 [14]. However, nanofiber could not be obtained from a single polymer substance without a polar group, such as PP, PE, etc. [17], although we have succeeded in fabricating PP nanofiber from PP/EVOH [27] and PP/PVB [28] blends. 

Polymer density is the main classifier of PE grades, and the first PE grade developed was low-density polyethylene (LDPE). LDPE is an excellent amorphous polymer with a relatively low melting point and good flow behavior, due to long side-chain branching [20]. As a result, it has advantages in the fabrication of a thin fiber using the laser melting process. Polyvinyl butyral (PVB) is a non-toxic, odorless, and environmentally friendly random amorphous copolymer [29,30,31], which is a very useful material in the electrospinning process [32]. PVB has been applied to potentially improve the spinnability of polymer solutions [33,34,35] and melts [28]. Accordingly, PVB could be a fruitful aid for PE nanofiber preparation that uses a laser melting device. The present work aims to produce low-density PE nanofibers from LDPE/PVB blended films using our line-like CO_2_ laser beam M-ESP device. In particular, we investigated the effect of PVB content on fiber spinnability, crystallinity, and diameter.

## 2. Experimental

### 2.1. Materials

A powder form of the blend PVB component, grade B60HH, comprised of 80–87% vinyl butyral, 12–16% vinyl alcohol, and 1–4% vinyl acetate, with a density (*ρ*) of 1.12 g/cm^3^, melting point (*T_m_*) of 140 °C, and a melt flow rate (MFR) of 14 g/10 min, was a gift from the Kuraray Co. Ltd., Tokyo, Japan. LDPE with MFR: 13 g/10 min, *T_m_*: 105 °C and *ρ*: 0.919 g/cm^3^ was the kind gift of the Tosoh Corp., Japan. Ethanol (99.5%) was also procured from the Nacalai Tesque, Inc., Kyoto, Japan.

### 2.2. Preparation of Blend Film

Various specimen blends were prepared with a kneading extruder (IMC-A300, Imoto Machinery Co., Ltd., Kyoto, Japan), by adding 10, 30, 50, 70, and 90 weight-% (wt.%) PVB to LDPE. Melt blending was conducted under N_2_ gas flow with a screw speed of 50 rpm at 190 °C for 10 min. Hereafter, film blend will be abbreviated as follows: a blended film comprising 50 wt.% PVB will be designated, PVB-50. A hot-press (Gonno Hydraulic Press Manufacturing Co., Osaka, Japan) was employed at 190 °C with a pressure of 25 MPa to fabricate LDPE/PVB sheets of 120 mm × 60 mm × 0.2 mm for preparation of nanofibers by laser M-ESP. Pure PVB and LDPE films were also made for comparison.

### 2.3. Formation of Fibers Using Laser M-ESP 

From LDPE/PVB blended film, fibers were fabricated by the M-ESP system equipped with a line-like CO_2_ laser melting device, at a room temperature of 25 ± 2 °C, and 68% ± 2% relative humidity; further details regarding the equipment were provided in our previous paper [27]. The blended film was automatically fed at a speed of 4 mm/min into the laser melting zone, where it was melted at around 200 °C by the laser beam. A high voltage was supplied to the copper electrode slit and laser power of 4.0 kV/cm and 40 W, respectively. A copper anode plate (150 mm × 150 mm × 5 mm) was placed 100 mm away from the electrode, and all electrospun fibers were collected on its surface. PVB was removed from as-spun LDPE/PVB blended fibers by ethanol treatment for 6.0 h at room temperature. For comparison, pure LDPE and PVB fibers were also prepared from the pure films following the same procedure.

### 2.4. Characterization

Differential scanning calorimetry (DSC) of produced films and fibers was carried out using DSC-60 (Shimadzu Corp., Kyoto, Japan). Samples were heated from 30 to 200 °C, held for 5 min, and cooled to 30 °C at a rate of (+) 10 °C and (−) 4 °C min^−1^, respectively. The crystallinity ($X_{c}\left(\%\right)$) of LDPE was calculated from the DSC results using the following equation [36]:

(1) $$X_{c}\left(\%\right)=\frac{\Delta H_{m}}{\Delta H_{m}{}^{o}\;\times \;{Ꞷ}_{PE}}\times 100.$$

where Δ$H_{m}$ is the experiential melting enthalpy of LDPE in the samples, Δ$H_{m}{}^{o}$ is the melting enthalpy of 100% crystallized PE (288 J g^−1^ [37]), and Ꞷ_PE_ is the weight fraction of LDPE in the compounds.

The rheological properties of samples were measured using a modular compact rheometer (MCR 302, Anton Paar, Graz, Austria). A parallel plate, 25 mm in diameter and with a gap of 1 mm was used. To determine the linear viscoelastic region, strain sweep experiments were conducted at a frequency of 1 rad s^−1^. Then, frequency sweep tests were carried out between 0.1 and 100 rad s^−1^ at a strain amplitude of 5%. All tests were performed at 190 °C under a nitrogen atmosphere. Disc polymer samples, 25 mm in diameter and 1-mm thick, were prepared using a hot press for tests.

Scanning electron microscopy (SEM) of fibers and film surfaces was carried out using a Keyence microscope (VE-9800; Keyence Co. Ltd., Osaka, Japan). An ion coater (SC-701; Sanyu Electron Co., Ltd., Tokyo, Japan) was used to gold-sputter coat the sample surface before SEM observation. Image analysis software (Photo Ruler) was used to measure the average and standard deviation of the PVB particle size in blend films and fiber diameters (indicated by *D* and *σ*), respectively, from the SEM images. Measurements were conducted for 3–5 SEM images of different areas of a sample, and more than 100 fibers were employed. Additionally, histograms were prepared for all individual sets of evaluations. 

The Fourier transform infrared (FTIR) spectrum was measured at room temperature within a range of 400–4000 cm^−1^ using an IR spectrometer (IR Affinity-1; Shimadzu Corp., Kyoto, Japan) equipped with a diamond/ZnSe crystal embedded single reflection ATR accessory (MIRacle 10; Shimadzu Corp., Kyoto, Japan). The characteristic band for pure PVB, pure LDPE, blended LDPE/PVB, and PVB-removed LDPE fibers were recorded.

## 3. Results and Discussion

### 3.1. Structural Analysis of LDPE/PVB Blend Films

**Figure 1:**
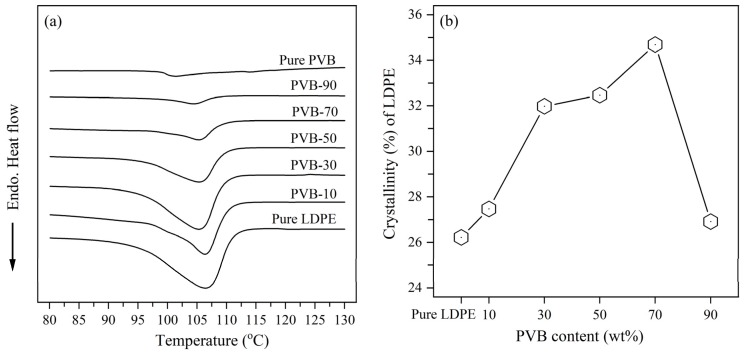
(**a**) Differential scanning calorimetry (DSC) heating curve of low density polyethylene/ polyvinyl butyral (LDPE/PVB) films; and (**b**) crystallinity (%) of LDPE in LDPE/PVB blends.

DSC measurements of the LDPE/PVB blend films verified the melting behavior of the compounds. Figure 1a contains DSC heating scans of LDPE/PVB blend films. Incremental increases of PVB fraction in blends caused a reduction of endothermic enthalpy (Δ$H_{m}$) of LDPE. Throughout the heat scans of LDPE/PVB blends, this influence is only from the crystalline polymer LDPE. As a result, the low ratios of LDPE in the blends ensured the decline of Δ$H_{m}$. In other words, relatively less energy was required to melt the compound with higher PVB content. Thus, the blending of PVB with LDPE resulted in the easy internal dispersion and thermal processing of the compounds.

From the DSC results, the crystallinity (%) of LDPE was calculated, as shown in Figure 1b. The addition of PVB greatly enhanced the crystallinity of LDPE. Without PVB, the observed crystallinity of LDPE was 26%; the inclusion of PVB raised this percentage, and the highest LDPE crystallinity (35%) was found for PVB-70. However, the maximum PVB content (90%) resulted in a slight reduction of crystallinity (27%). Reza Zanjanijam et al. [36] found the surface of the PVB domain acts as a nucleating agent for PP, which improved the structure of the PP crystal and resulted in an increase of crystallinity. PP and PE are members of the same polyolefin family and show a similar interaction with PVB. Consequently, the crystallinity of LDPE was increased by the inclusion of PVB in LDPE/PVB blends. In contrast, as discussed in the SEM images of blend films, the structure of the LDPE/PVB blend with 90% PVB was damaged in ethanol solution due to the elimination of the large amount of PVB from the compound (Supplementary Materials, Section 1, Figure S1). Therefore, a little deterioration and puffiness of the LDPE crystal occurred for large PVB particles inside, resulting in the reduction of LDPE crystallinity increments for PVB-90 blends. Even so, the high PVB-mixed LDPE showed slightly higher crystallinity than pure LDPE, due to the nucleating effect of PVB. The inclusion of PVB also improved the tensile strength of LDPE/PVB film (Supplementary Materials, Section 2, Figure S2), which indicates the better molecular arrangement of LDPE. It can thus be said that the structure of LDPE crystal in LDPE/PVB blends is undoubtedly improved by PVB. 

### 3.2. Rheological Properties of Blends

**Figure 2:**
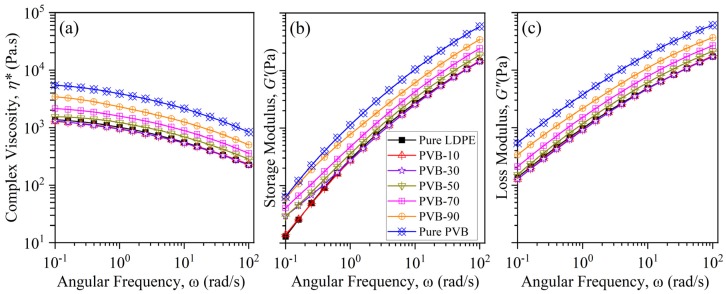
(**a**) Complex viscosity; (**b**) storage; and (**c**) loss moduli as a function of angular frequency for pure LDPE, pure PVB and LDPE/PVB blends.

Figure 2 illustrates the linear viscoelastic properties of the LDPE/PVB blends, including pure LDPE and PVB. The complex viscosity, storage, and loss moduli of pure PVB were significantly higher than those of pure LDPE. As a result, the addition of PVB increased the complex viscosity of LDPE/PVB blends. For the storage and loss modulus of blends, similar characteristics were apparent. However, the viscoelastic behavior of PVB-10 and PVB-30 were very close to pure LDPE, due to the low amount of PVB in the blend. In contrast, the blend with 90% PVB (PVB-90) showed viscosity close to that of pure PVB. LDPE and PVB are totally immiscible, so adding PVB enhances the viscosity of the blends proportionally.

### 3.3. Structure of As-Spun LDPE/PVB Blend Fibers

**Figure 3:**
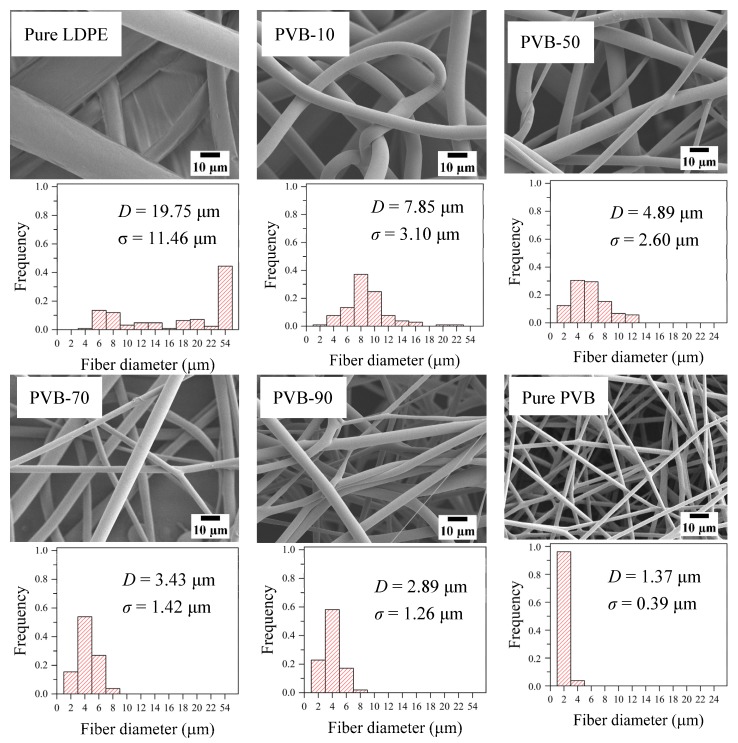
SEM images and histograms of as-spun pure LDPE and LDPE/PVB blend fibers.

The SEM images of as-spun pure LDPE, pure PVB, and LDPE/PVB blend fibers are shown with histograms in Figure 3. PVB had a substantial impact on easing the M-ESP process and minimizing the as-spun fiber diameter. The electrospinning of pure LDPE by the laser melting device was very tough, and a few of Taylor cones were developed with a larger dimension. Hence, a very large (*D* = 19.75 µm) diameter with a higher standard deviation (*σ* = 11.46 µm) was found for pure LDPE fiber. The addition of PVB increased the number of Taylor cones per cm (Supplementary Materials, Section 3, Figure S3) and decreased the fiber diameter with increasing uniformity. Even the addition of only 10% PVB with LDPE resulted in a smoother electrospinning process and fiber with a 7.86-µm diameter. Greater inclusion of PVB resulted in a further decrease of fiber diameter and better uniformity. Consequently, the highest PVB dosing (90%) resulted in the lowest diameter (2.89 µm) of LDPE/PVB blend fiber, very close to the fiber produced from pure PVB (1.37 µm). 

As mentioned in the DSC section, PVB inclusion eased the thermodynamic process by prompting interior molecular dispersion in blends. In addition to having an outstanding surface adhesion property [38,39], PVB molecules encourage the heterogeneous nucleating activity for polyolefins during high-temperature processing [36]. As a result, the LDPE molecules were easier to handle during M-ESP, enabling well-drawn long polymer chains. Thus, the blended polymer jet was regularly discharged from the Taylor cone with a thinner dimension. In Section 3.2 on rheological properties, we observed that the complex viscosity of blends increased proportionally with PVB dosing. Whereas the higher viscosity of polyolefins is considered the major barrier to its fiber diameter minimization, here, the polarity of PVB played an important role in the reduction of as-spun fiber diameter by preventing the complications of higher viscosity blends. The polarity and the immiscibility of PVB with LDPE increased the electrostatic attraction of the grounded collector to the melted polymer compound, resulting in a significant reduction in fiber diameter with better spinnability (Supplementary Materials, Section 3).

### 3.4. Structure of PVB-Removed LDPE Fibers

**Figure 4:**
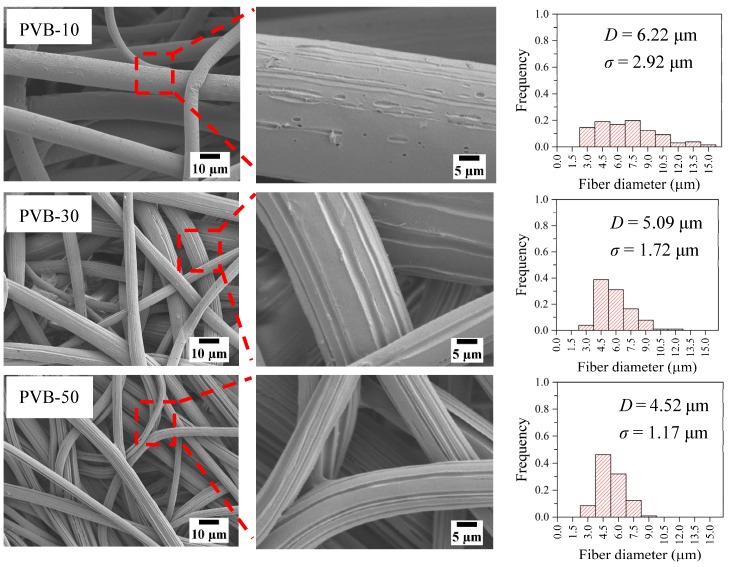
SEM images and histograms of PVB-removed LDPE/PVB blend fibers.

SEM micrographs of PVB-removed LDPE fibers obtained from LDPE-rich LDPE/PVB blend fibers are shown in Figure 4. Parallel grooves from where the PVB was removed were visible on the LDPE fiber surface for PVB-30 & 50, while some holes with irregular channels were also found in PVB-10. The parallel arrangement of clear PVB-removal grooves confirmed the excellent surface adhesion properties of PVB [38,39], including the improved drawing effect during M-ESP. As may be seen, the PVB removal channels became deeper and more pronounced with increasing increments of PVB in blends. In addition, PVB elimination from LDPE-rich samples also decreased the ultimate fiber diameter. However, the deep channels on the fiber surface left by PVB removal, rambled the geometric shape of the cross-section. 

**Figure 5:**
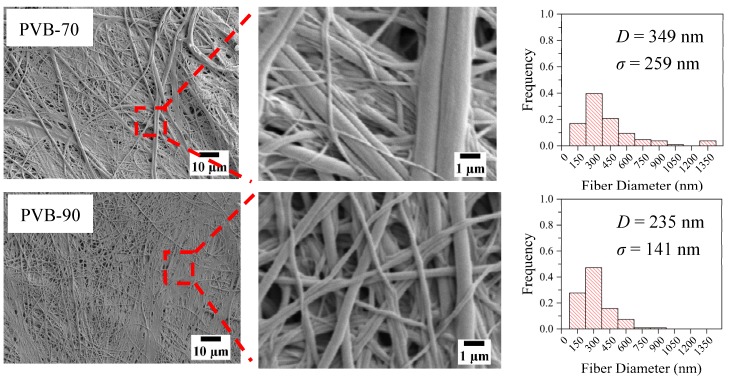
SEM images and histograms of PVB-removed LDPE/PVB blend fibers.

The crucial outcome of this study was revealed after PVB was removed from the PVB-rich samples shown in Figure 5. A massive drop in the LDPE fiber diameter was observed, due to PVB removal from PVB-rich specimens, particularly PVB-70 and -90 blend fibers. Eliminating PVB from LDPE-rich samples produced the multiple parallel grooves on the fiber surface that are shown in Figure 4. For PVB-rich samples, these grooves were deeper, finally resulting in the splitting of single or multiple fibers. Consequently, the LDPE nanofibers with a diameter of 349 ± 259 nm and 235 ± 141 nm were found for PVB-70 and 90, respectively. Although the 90% PVB blend fiber was confirmed to produce more delicate LDPE nanofiber with better consistency, PVB-70 also created LDPE fibers on a nanoscale dimension while using less PVB.

**Figure 6:**
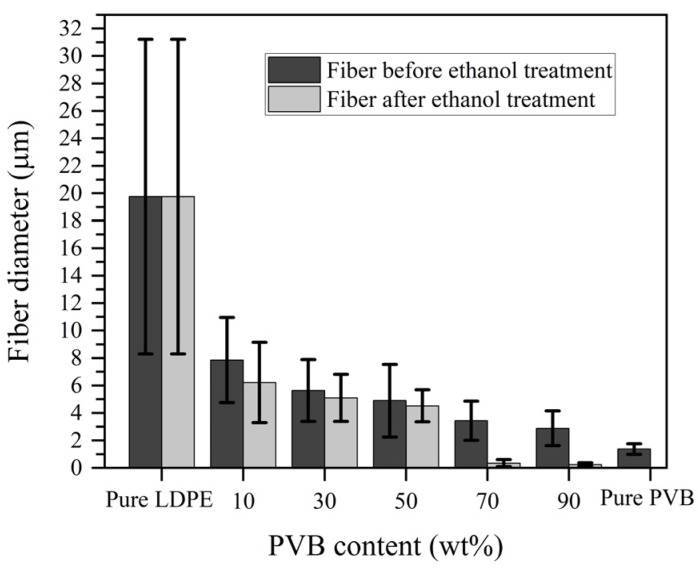
Comparison of the diameters of LDPE/PVB blends and PVB-removed LDPE fibers.

Figure 6 compares the fiber diameter of LDPE/PVB blends and PVB-removed LDPE fibers. A two-step fiber diameter reduction was observed, first during M-ESP with PVB and LDPE mixtures; and second when PVB was removed from LDPE/PVB blend fibers. Due to the addition of PVB, a massive diameter drop of blend fiber was observed for PVB-10, which then continued linearly up to the PVB-90. Slight and huge fiber diameter drops were observed when PVB was removed from LDPE- and PVB-rich samples, respectively. A significant reduction in fiber diameter was found for the PVB-90 blend fiber, from 2.89 ± 1.26 µm to 235 ± 141 nm due to the multiple splitting of LDPE fibers. The histograms and standard deviations also confirmed that more consistent fibers were obtained with higher PVB content. Most of the PVB-rich samples were formed by PVB. Hence, ethanol removed a substantial portion of the fiber, leaving only 10% LDPE, which demonstrated more delicate LDPE nanofibers. Furthermore, the development of multiple single nanofibers, due to fiber splitting, ensured a huge drop in fiber diameter. Therefore, nanofibers with minimal diameter were also obtained; this, however, caused an extended standard deviation.

### 3.5. DSC Analysis of Fibers

**Figure 7:**
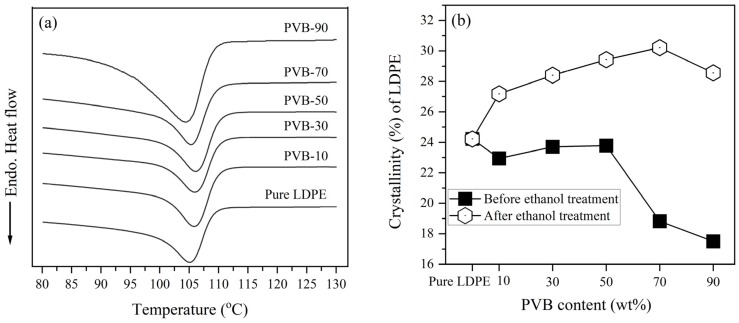
(**a**) DSC heat inspections of PVB removed LDPE and pure LDPE fibers; and (**b**) crystallinity (%) of melt electrospun fibers before and after ethanol treatment.

Figure 7 shows DSC heat values of PVB-removed LDPE and pure LDPE fibers and the crystallinity of as-spun fibers before and after ethanol treatment. PVB-removed LDPE fibers exposed the melting curve like pure LDPE fiber, but exhibited extended endothermic slope magnitude. In Section 3.1, Figure 1b showed that including PVB truly improved both the crystal structure and crystallinity of LDPE. However, the laser M-ESP process had a detrimental effect on the crystallinity of pure LDPE and blend polymers. The crystallinity of LDPE was drastically decreased for electrospinning in blends with higher PVB content. Generally, the crystallinity of a polymer is a function of the supercooling [40,41] and is significantly lower for electrospun fibers compared to samples prepared by common processing technologies (molding or film casting) [42]. Here, M-ESP was conducted at room temperature, where the polymer was melted at around 200 °C by laser, and as-spun fibers were collected immediately by a collector. As a result, the melted electrospun blend fiber was quenched and solidified very quickly before the crystal formed properly, degrading crystallinity. The thinner fibers cooled more rapidly, and resulted in a greater reduction of crystallinity for PVB-rich blend fibers. Instead, after ethanol treatment, the crystallinity of as-spun fiber was regained and reached near the value of blend films. 

The highest improvement was observed after the ethanol treatment for PVB-rich samples. For PVB-90, crystallinity was recouped from 18% to 27%. The ethanol treatment was applied to as-spun LDPE/PVB blend fibers at room temperature for 6 h. To remove PVB from closely associated LDPE, ethanol enters the core of the LDPE fiber and enhances the arrangement of polymer chains after the PVB matrix dissolves. Polaskova et al. [43] also observed the improved molecular orientation and crystallinity of electrospun fiber with accompany of methanol solution. Like LDPE/PVB film, the crystallinity of PVB-removed LDPE fiber was improved by the increase of PVB content in blend fibers. The highest crystallinity (30%) was found for PVB-70, while only 24% of crystallinity was recorded for pure LDPE fiber. We found the finest LDPE nanofiber from the PVB-90 blend demonstrated crystallinity of around 28%, which is also higher than the crystallinity of pure LDPE fiber. Thus, we confirmed that LDPE fiber prepared from LDPE/PVB blends showed better crystallinity than that made from pure LDPE. 

### 3.6. FTIR Analysis of Fibers

**Figure 8:**
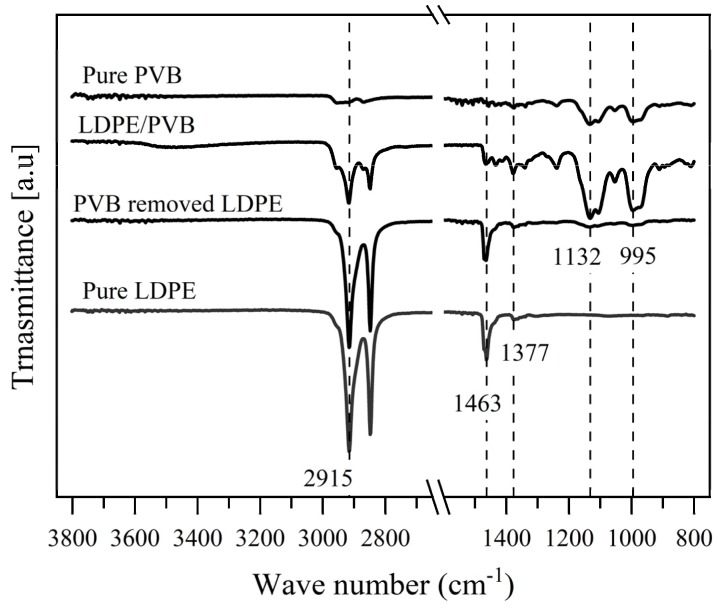
Fourier transform infrared (FTIR) spectrum of pure PVB, pure LDPE, PVB-removed LDPE, and LDPE/PVB blend fibers.

Figure 8 shows the FTIR spectra of pure PVB, pure LDPE, PVB-removed LDPE, and LDPE/PVB blend fibers. Peaks at 1132 cm^−1^ and 995 cm^−1^ corresponding to a C-O-C butyral ring and C-O stretching [44], respectively, are the typical bands of PVB. Both the LDPE/PVB blended and pure PVB fibers spectrums strongly revealed those characteristic peaks. However, after ethanol treatment, the peaks disappeared from the LDPE/PVB blended fiber. This confirmed the complete elimination of PVB from blended fiber by ethanol treatment. Other typical bands at 1377 cm^−1^ (distinguishing band for polyolefin [45]), 1463 cm^−1^, and 2915 cm^−1^ ascribed to -CH_3_ symmetric deformation vibrations, bending deformation, and -CH_2_ asymmetric [46], respectively, are firmly visible for pure LDPE and PVB-removed LDPE fiber. This supported the notion that the characteristics of PVB-removed LDPE and pure LDPE fiber are similar. We concluded that the melt electrospun LDPE fiber from LDPE/PVB blend film is a pure LDPE fiber. 

## 4. Conclusions

We successfully minimized the fiber diameter of LDPE nanofibers, producing fibers that are a few hundred nanometers in diameter by laser M-ESP system, using PVB as a blend component. Here, the process involved two-step fiber diameter reduction: during M-ESP via the inclusion of PVB and by subsequently removing PVB from LDPE/PVB blend fiber. A massive blend fiber diameter drop was observed with even a low 10% inclusion of PVB, and the trend continued linearly up to 90% PVB content. In addition, PVB improved the polarity and drawing effect of LDPE/PVB blend films during laser melting, which facilitated the production of a more delicate fiber with better spinnability. A further drop of LDPE fiber diameter was achieved after PVB was removed from LDPE/PVB blend fibers, due to the fiber splitting, mostly in PVB-rich samples. Consequently, LDPE nanofibers with a diameter of 235 ± 141 nm were obtained from blended fiber using 90% PVB. DSC results also revealed that the improved crystallinity of LDPE in the film was decreased for M-ESP but regained after ethanol treatment. These superfine polyethylene nanofibers constitute a promising material for battery separators and filtration media for waste-water treatment.

## Supplementary Materials

The following are available online at https://www.mdpi.com/2073-4360/12/2/457/s1, Figure S1: SEM micrographs of blend films surface after removing PVB from (a) PVB-10, (b) PVB-30, (c) PVB-50, (d) PVB-70, (e) relation of particle size of the PVB domains, Figure S2: Tensile stress-strain curve of (a) pure LDPE, pure PVB, and (b) LDPE/PVB blend films, Figure S3: Images and the number of Taylor cones during laser M-ESP from different blend films.
