# Supplementary material for: Melt-Electrospun Polyethylene Nanofiber Obtained from Polyethylene/Polyvinyl Butyral Blend Film
Source: Polymers (Basel). 2020 Feb 16;12(2):457. doi: 10.3390/polym12020457 (PMC7077628; doi:10.3390/polym12020457)
Supplement: Supplementary file 1 [file polymers-12-00457-s001.pdf]

Supplementary Material

Melt-electrospun polyethylene nanofiber obtained from polyethylene / polyvinyl butyral blend film

Mohammad Zakaria[1,2], Kanta Shibahara[1] Koji Nakane[1,*]

[1]Frontier Fiber Technology and Science, University of Fukui, Fukui 910-8507, Japan.

[2]Department of Textile Engineering, Dhaka University of Engineering & Technology, Gazipur 1700, Bangladesh. Email: zakariate@duet.ac.bd

*Correspondence to: Koji Nakane (E-mail: nakane@matse.u-fukui.ac.jp)

Contents

Section 1. PVB particle size in LDPE/PVB blend film

Section 2. Mechanical properties of LDPE/PVB blend film

Section 3. Spinnability of as-spun fiber from LDPE/PVB blend film by laser melt electrospinning

Section 1. PVB particle size in LDPE/PVB blend film

The size of PVB particles in blend film was assessed and displays in Figure S1 (e). An incremental trend of PVB particle size was found with the addition of PVB in blends. The PVB particle size for PVB-10 was around 1.55 μm and increased up-to 9.4 μm for PVB-70. And the film structure of the LDPE/PVB blend with 90% PVB collapsed in the ethanol solution due to the elimination of large-sized PVB from the compound. As observed, the distribution and size of PVB particle in blends are more irregular and also increase with the addition of PVB content. Based on the SEM illustrations, it could be found that the addition of a small amount (10%) of PVB increased the size of PVB particles. We concluded there was immiscibility or very weak attraction between LDPE and PVB molecules. Reza Zanjanijam et. al. [SR1] observed a similar morphological structure in PP/PVB blends and remarked on the immiscibility between the dispersed and matrix phases as a result of weak adhesion between PP and PVB.

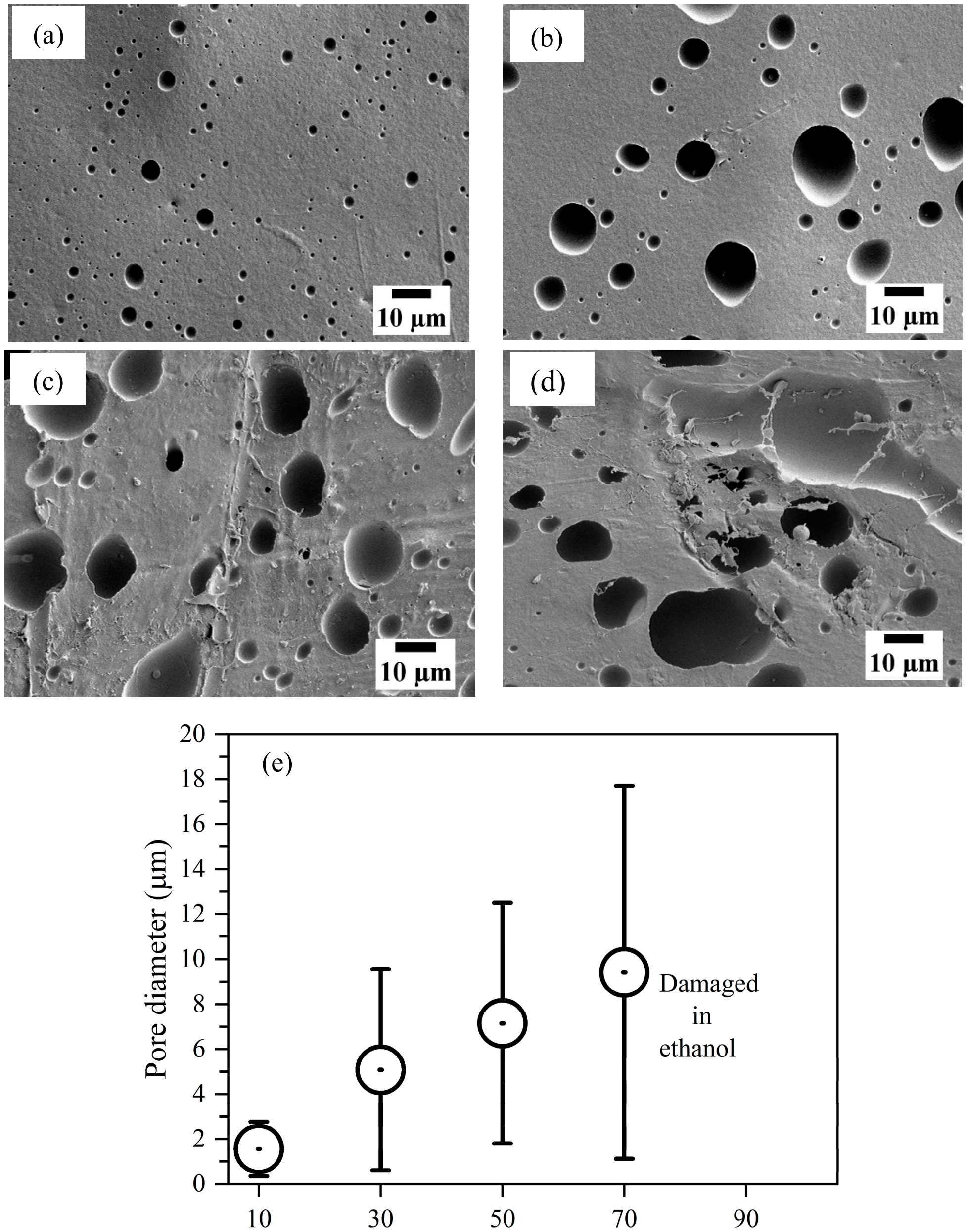

Figure S1: SEM micrographs of blend films surface after removing PVB from (a) PVB-10, (b) PVB-30, (c) PVB-50, (d) PVB-70, (e) relation of particle size of the PVB domains.

Section 2. Mechanical properties of LDPE/PVB blend film

The tensile test for the polymer films was performed at room temperature with a Tensilon UTM-III tensile tester (Toyo Baldwin, Japan). For the tensile testing, the sample with 50 mm (sample width, 10 mm) initial gauge length was held between two clamps. The bottom clamp was connected to a cross-head that moves downwards at a preprogrammed rate of 50 mm/min for applying tension to the sample. A load cell was positioned to the upper clump which recorded the applied tension to the sample. And the displacement was measured from the crosshead position. The tensile stress-strain curves of pure LDPE, PVB and LDPE/PVB blend films were calculated and showed in Figure S2. The highest tensile strength was observed for pure PVB and the pure LDPE displayed the maximum strain. For LDPE/PVB blend films, with the increase of PVB content the tensile strength was increased with reduced flexibility. Unsuitably, PVB-50 exhibited less tensile strength than PVB-30 and PVB-10. The improvement of tensile strength for PVB rich film indicates the better molecular arrangement of LDPE due to the plasticizing effect of PVB [SR1]. PVB is well-known as a tough material and LDPE has a great extent of flexibility against tensile loading. But, the combined perfection of toughness and flexibility was inexistent for LDPE/PVB blend due to the thermodynamic immiscibility of LDPE and PVB.

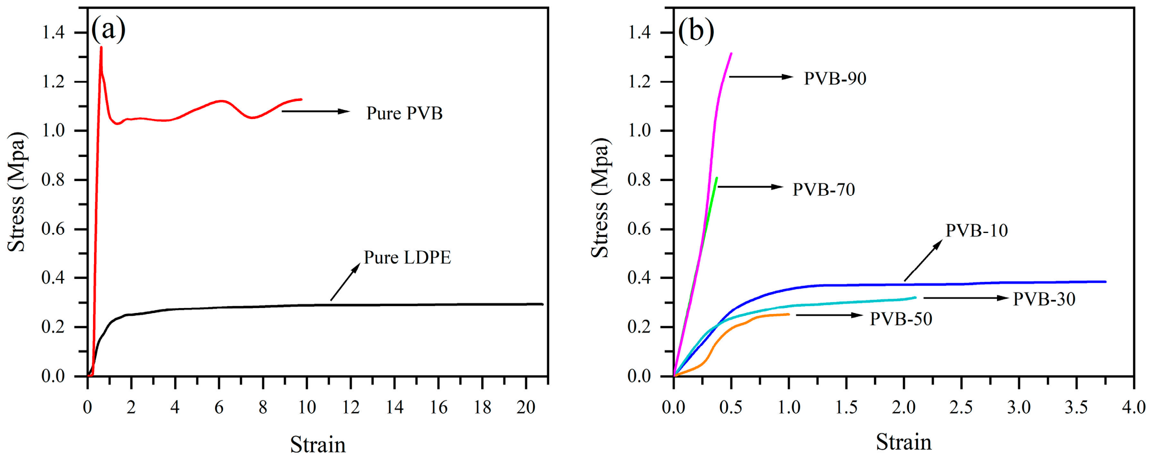

Figure S2: Tensile stress-strain curve of (a) pure LDPE, pure PVB, and (b) LDPE/PVB blend films

Section 3. Spinnability of as-spun fiber from LDPE/PVB blend film by laser melt electrospinning

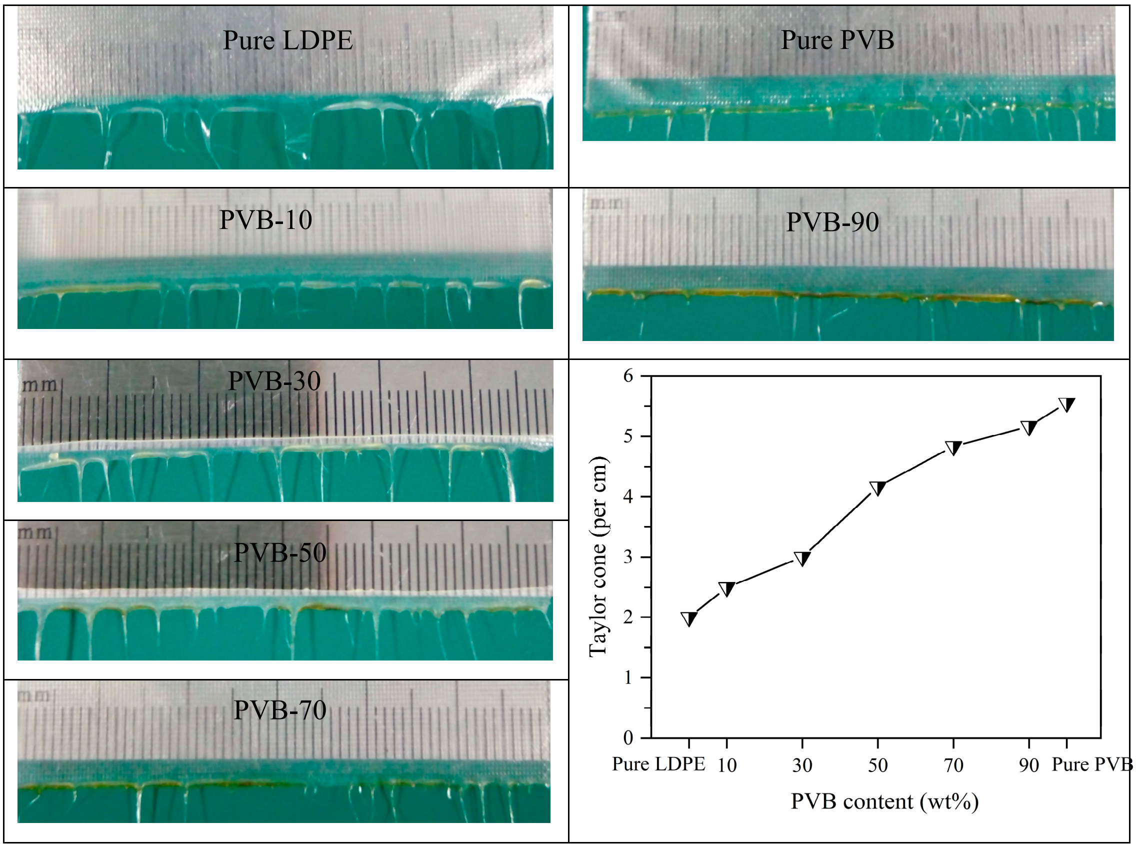

Figure S3: Images and the number of Taylor cones during laser M-ESP from different blend films

Figure S3 illustrate the images and the number of Taylor cone developed in laser melted film for different PP/PVB blends during electrospinning. Electrospinning of pure LDPE by laser melting device is very tough, and a few numbers of Taylor cones were observed with a larger dimension. The Taylor cones are not evenly distributed, and some undeveloped cones can be seen, mostly in LDPE rich samples. For pure LDPE and PVB-90, only 2.00 and 5.17 Taylor

cones per cm were developed, respectively. With the increase of PVB content in blends, the number of Taylor cones enhanced. Which reveals the better spinnability of fiber from PVB rich LDPE/PVB blend than pure LDPE. Wang et al. [SR2] also commented that the high viscosity and low conductivity of PP restrict the smooth production of fiber by M-ESP. The surface color of the Taylor cone formed from LDPE/PVB blends had a tendency to turn black due to the thermal oxidation of PVB at high temperature, which is absent for pure LDPE. Merinska et al. also observed the yellow (or sometimes brown) color of PVB due to the thermo-oxidative reaction during its reproduction at >160 of temperature [SR3].

References

SR1. Reza Zanjanijam, A.; Hakim, S.; Azizi, H. Morphological, dynamic mechanical, rheological and impact strength properties of the PP/PVB blends: the effect of waste PVB as a toughener. *RSC Adv.* **2016**, *6*, 44673–44686.

SR2. Wang, D.; Sun, G.; Chiou, B.-S.; Hinestroza, J.P. Controllable fabrication and properties of polypropylene nanofibers. *Polym. Eng. Sci.* **2007**, *47*, 1865–1872.

SR3. Měřínská, D.; Tupý, M.; Kašpárková, V.; Popelková, J.; Zvoníček, J.; Pištěk, D.; Svoboda, P. Degradation of Plasticized PVB During Reprocessing by Kneading. *Macromol. Symp.* **2009**, *286*, 107–115.
